# Novel SNP markers and other stress-related genomic regions associated with nitrogen use efficiency in cassava

**DOI:** 10.3389/fpls.2024.1376520

**Published:** 2024-04-04

**Authors:** Joseph Okpani Mbe, Daniel Kwadjo Dzidzienyo, Simon Peter Abah, Damian Ndubuisi Njoku, Joseph Onyeka, Pangirayi Tongoona, Chiedozie Egesi

**Affiliations:** ^1^ Cassava Research Program, National Root Crops Research Institute (NRCRI), Umudike, Nigeria; ^2^ West Africa Centre for Crop Improvement (WACCI), University of Ghana, Accra, Ghana; ^3^ Biotechnology Centre, College of Basic and Applied Sciences, University of Ghana, Accra, Ghana; ^4^ Department of Plant Breeding and Genetics, Cornell University, Ithaca, NY, United States

**Keywords:** cassava, genome-wide association studies, nitrogen use efficiency, SNP, marker

## Abstract

Cassava productivity is constrained by low soil nitrogen, which is predominant in most cassava-growing regions in the tropics and subtropical agroecology. Improving the low nitrogen tolerance of cassava has become an important breeding objective. The current study aimed to develop cassava varieties with improved nitrogen use efficiency by identifying genomic regions and candidate genes linked to nitrogen use efficiency in cassava. A genome-wide association study (GWAS) was performed using the Genome Association and Prediction Integrated Tool (GAPIT). A panel of 265 diverse cassava genotypes was phenotyped for 10 physiological and agronomic traits under optimum and low-nitrogen regimes. Whole-genome genotyping of these cassava cloneswas performed using the Diversity Arrays Technology (DArTseq) sequencing platform. A total of 68,814 single nucleotide polymorphisms (SNPs) were identified, which were spread across the entire 18 chromosomes of the cassava genome, of which 52 SNPs at various densities were found to be associated with nitrogen use efficiency in cassava and other yield-related traits. The putative genes identified through GWAS, especially those with significant associated SNP markers for NUE and related traits have the potential, if deployed appropriately, to develop cassava varieties with improved nitrogen use efficiency, which would translate to a reduction in the economic and environmental cost of cassava production.

## Introduction

Cassava (*Manihot esculenta* Crantz) is among the most produced commodity crops in Nigeria, and its demand is on the rise, especially now that it is considered as an alternative to wheat. Meeting the demand of the growing population in the region calls for urgent attention, as the yield trend (production per hectare) is still low (Poverty Oxford and Human Development Initiative, 2017; FAO, 2018). This low yield is usually linked to a decline in soil fertility and limited resources for inputs such as fertilizers. However, there is a growing concern that the booming agro-allied industries, which rely heavily on cassava as a raw material, may adversely affect the continued availability of cassava-based products to Nigerian families. Soil fertility decline, caused by high rainfall, erosion, leaching, overgrazing, bush burning, and biodiversity loss, etc., is a major constraint faced by farmers in savanna zones of the world. Soil nutrient depletion, especially nitrogen, together with biotic and abiotic stresses, are the major reasons for the low productivity of cassava in sub-Saharan Africa. Nitrogen deficiency affects stay-green ability, photosynthetic rate, translocation of organic compounds from source to sink, root bulking, and root yield (De Souza et al., 2017). Nitrogen is an important nutrient element required by plants to attain their genetic yield potential. Recently, crop land around the world has received 119.41 million tons of nitrogen fertilizer to produce the desired yield (FAO, 2018).

According to the International Fertilizer Association (IFA, 2022), the demand for nitrogen fertilizer is expected to increase by 1.1% annually by 2021. As urea constitutes most of the newly added nitrogen capacity, the demand for urea is expected to grow at a faster rate. Unfortunately, the widespread use of nitrogen fertilizers has had detrimental impacts on the ecosystem, including pollution and ecological imbalances (Miller and Cramer, 2004). There are three key components of nitrogen absorption by plants: uptake, assimilation, and remobilization (Han et al., 2016). Several genetic and physiological mechanisms govern these processes. The efficiency of nitrogen utilization is defined as the capacity of the cassava genotype to grow, develop, and reproduce in the presence of nitrogen nutritional deficits in the soil (Fritsche-Neto et al., 2010). The product of nitrogen uptake efficiency (NUpE) and nitrogen utilization efficiency (NUtE) is nitrogen use efficiency (NUE) (Good et al., 2004). Reports have shown that increased NUE has a significant positive correlation with crop biomass and yield (Stanley et al., 2006; Wu et al., 2016), suggesting that selection based on NUE can improve cassava productivity. Kant et al. (2011) also claimed that a 1% improvement in NUE may result in annual savings of $1.1 billion.

However, in the genetic improvement of nitrogen use efficiency, the first step is to determine the amount of genetic variability within the plant population. To isolate the contribution of different nitrogen regimes or levels from genetics and other environmental factors to cassava root yield, it is necessary to evaluate the plant population under low and optimum nitrogen conditions. However, there are other factors that will come into play apart from genetics, such as radiation use efficiency (De Souza et al., 2017), the interplay between macro- and micronutrients, and the interaction between water availability and nitrogen uptake by plants (Mei et al., 2015). Genome-wide association studies (GWAS) are an effective method for discovering new genes and validating suspected genes involved in complex phenotypic characteristics (Altshuler et al., 2008). GWAS have been used to map the key genes for different cassava characteristics. Among the parameters that have been studied include the genetic makeup of cassava mosaic disease (Wolfe et al., 2016), beta-carotene content, dry matter content, shoot weight, fresh root yield, starch yield, starch fraction amylose content, and dry matter content (De Oliveira et al., 2012). This work aims to contribute to the development of cassava varieties with improved nitrogen use efficiency, which will translate to a reduction in the economic and environmental costs of cassava production. The main objective of this study was to identify SNP marker loci that are strongly associated with fresh root yield and other secondary characteristics under low and optimal N regimes. Other objectives included: (i) identifying candidate genes linked to nitrogen utilization efficiency and other related traits, (ii) identifying the best performing test clones under optimum and low nitrogen regimes, and (iii) assessing the population structure among the clones.

## Materials and methods

### Study sites, germplasm, and field trials

The studies were conducted in three major cassava-growing regions in Nigeria with distinct climatic attributes (**Table 1**). Before land preparation, composite auger soil samples were taken from the experimental sites at soil depths ranging from 0 cm to 20 cm in both years across locations. The soil samples were air-dried, crushed, thoroughly mixed, and passed through a 2-mm sieve before subjecting the soils to routine soil analysis as described by Udo and Ogunwale (1978) for the determination of nitrogen, phosphorus, and potassium (N.P.K). The data derived from soil analysis are presented in **Table 2**.

**Table 1:** Coordinates and other attributes of the study locations.

Location	Environment	Coordinate	Altitude	Temperature	Rainfall
Umudike	Humid forest	7° 24″ E, 5o 29″ N	120 m	22°C to 31°C	2,200 mm
Igbariam	Forest savanna	7° 31″ E, 5° 56″ N	150 m	24°C to 32°C	1,800 mm
Otobi	Guinea savanna	7°20″ E, 8°41″ N	319 m	24°C to 35°C	1,500 mm

**Table 2:** Soil fertility status (N.P.K) in Umudike, Igbariam, and Otobi.

Location	Nitrogen N (%)		Potassium P (pmm)		Potassium K (me/mg)	
Year	2020	2021	2020	2021	2020	2021
Umudike	0.14	0.12	27	25	0.40	0.37
Igbariam	0.07	0.10	16	15	0.05	0.08
Otobi	0.15	0.13	22	24	0.30	0.17

A panel of 260 diverse cassava genotypes and five (5) checks was used in these studies. The clones were NEXTGEN cassava materials from the International Centro Internacional de Agricultura Tropical (CIAT), the International Institute for Tropical Agriculture (IITA), and the National Root Crops Research Institute (NRCRI) Umudike Nigeria. The clones were evaluated during the 2020–2021 and 2021–2022 planting seasons in three environments (Umudike, Igbariam, and Otobi) in Nigeria, using an augmented block design. A weather logger was installed across the environment to collect climate data. Plot sizes of 1 m × 0.8 m, were adopted giving a total plant population of 12,000 plants per hectare. The optimum and low-nitrogen regimes were separated by a 0.5 m spacing distance.

Soil fertility classes and cassava N.P.K recommendations for each class in Nigeria, as described by Chude et al. (2012), were used as a guide in fertilizer application (**Table 3**).

**Table 3:** Soil fertility classes and cassava N.P.K recommendation for each class.

Nutrients	Low	Medium	High	Reference
**Nitrogen N (%)** *Recommendation*	<0.15 *196 kg/ha*	0.15–0.20 *98 kg/ha*	>0.20 *49 kg/ha*	Chude et al. (2012)
**Potassium P (pmm)** *Recommendation*	<15 *288 kg/ha*	15–25 *144 kg/ha*	>25 *72 kg/ha*	Chude et al. (2012)
**Potassium K (me/mg)** *Recommendation*	<0.15 *90 kg/ha*	0.15–0.25 *45 kg/ha*	>0.25 *0 kg/ha*	Chude et al. (2012)

### Phenotyping

An association panel of 265 cassava genotypes was phenotyped for the following qualitative and quantitative traits: plant vigor, stay green ability, leaf retention, photosynthetic active radiation, leaf area index, chlorophyll and leaf nitrogen, fresh root yield, specific gravity, dry matter content, dry root yield harvest index, and starch content using IITA standard operation practice and NEXTGEN cassava breeding ontology.

### Data analyses

Using the lme4 (Linear Mixed-Effects Models utilizing “Eigen” and “S4” package implemented in R software), three experimental years’ phenotypic data were initially examined independently and then pooled across years.

Analyses of variance within and across environments for each population under each management condition were conducted using the R program integrated within the META-R software (Alvarado et al., 2015). Using the linear mixed model, the following variance components were determined,


$$Y_{ijk}=\mu +g_{i}+l_{j}+\tau_{ij}+\beta {\left(c\right)}_{ik}+\varepsilon_{ijk}$$

where $Y_{ijk}$ was the phenotypic performance of the *i*th genotype at the *j*th environment in the *i*th unreplicated genotypes nested within the *k*th replicated checks, µ was population mean, gi was the genetic effect of the *i*th genotype, lj was the effect of the *j*th environment, $\tau_{ij}$ was the interaction of *i*th genotypes and the *j*th environment, $\beta {\left(c\right)}_{ik}$ was the effect of the *i*th unreplicated genotypes nested within the *k*th replicated checks, $\varepsilon_{\text{ijk}}$ was the residual.

Environment and genotype were classified as random effects, whereas checks nested in the blocks were treated as fixed. The ratio of genotypic to phenotypic variance was used to estimate broad-sense heritability on an entry-mean basis. Moreover, BLUP was calculated for all attributes of each clone across all environments and management.

### Estimation of nitrogen use efficiency

Most root and tuber crops (RTCs), including potatoes, have been studied for their nitrogen use efficiency (NUE) (Sharifi et al., 2007; Zebarth et al., 2008). However, the definition of NUE and the units employed in its calculation vary among studies (Tiwari et al., 2018). However, spanning across numerous crops, nitrogen use efficiency (NUE) is defined as:


$$\text{NUE}=\frac{\text{Yield}}{\text{Available N}}$$

Yet, each crop will define “yield” differently. The “yield” in relation to potatoes, cassava, and other root and tubers can include root wet weight, root dry weight, or whole plant dry weight (Tiwari et al., 2018). Each of these categories has limitations: plant weight and dry weight do not accurately represent edible or marketable yield, whereas tuber wet weight varies with environmental influences.

According to Kratzke and Palta (1985), tuber-specific root structures may also be responsible for tubers water intake, and water stress can cause changes in root water content (Levy et al., 2013). To eliminate any confounding variables, gain insight into the phenotypic determinants of yield, and be consistent with current work in the field, root dry matter was employed in this study as the numerator for NUE (Getahun, 2017).

The two components of nitrogen use efficiency (NUE), nitrogen utilization efficiency (NUtE), and nitrogen uptake efficiency (NUpE) are typically modeled as a product (Moll et al., 1982; Bock, 1984). The relationships between NUE, NutE, and NUpE are typically represented as


$$\begin{array}{l} \;\left(NUE\right)\;\;\;\;\;\;\;\;\;\;\;\left(NUtE\right)\;\;\;\;\;\;\;(NUpE)\\ \frac{Tuber\;Dry\;matter}{Applied\;N}=\frac{Tuber\;Dry\;matter}{Plant\;N}x\frac{Plant\;N}{Applied\;N} \end{array}$$

Applied N is the standard denominator of NUE studies in root and tuber crops, such as potatoes (Tiwari et al., 2018) and cassava (Liang et al., 2020). In the present study, the assessment of 265 cassava genotypes showed significant differences in nitrogen use efficiency under different N treatments. A mixed model-derived best linear unbiased prediction selection index was used to evaluate the performance of the clones under optimum and low nitrogen regimes. Due to the number of clones used, NUE was categorized into five different levels: very high, high, moderate, low, and very low NUE.

### Genotyping and DArTseq SNP filtering

The genomic DNA of 265 diverse cassava genotypes was extracted from fully expanded cassava leaves using the CTAB method as described by Huang et al. (2000). The DNA concentration and purity were checked using a NanoDrop 2000 spectrophotometer (ND—2000 V3.5, NanoDrop Technologies, Inc.) before it was shipped to the Diversity Arrays Technology (DArT) Pty. Ltd., Australia, (http://www.diversityarrays.com/dart-mapsequences) for whole-genome sequencing using the DArTseq platform.

On the platform created by Cruz et al. (2013), whole-genome genotyping of 265 cassava clones was performed utilizing 68, 814 DArT markers. A consensus DArT map was used to infer the marker order and position, which was then used to integrate the markers into a linkage map. With a repeatability index of 0.93, the mean polymorphic information content was 0.16 and ranged from 0.0 to 0.50.

Based on Mwadzingeni et al. (2017), DArTseq SNP-generated markers were filtered by imputation to exclude problematic SNPs with >5% of missing data. The 68, 814 silico DArT markers distributed across 18 chromosomes were used to genotype every individual. After imputing the missing values, 63,362 DArTseq markers were used in the analysis.

### Linkage disequilibrium and population structure

For the 265 cassava clones, the alleles per locus, allele frequency, and anticipated heterozygosity were estimated to quantify the genetic variation. The genetic distances of the genotypes were estimated. In accordance with Pritchard et al. (2000), the population structure was created with the aid of the Bayesian clustering approach in the structure version 2.3 programs, and genetic clusters were generated using the adjacent joining approach. Based on 63,362 DArTseq-derived SNP markers dispersed throughout the cassava genome, the population structure was determined using 10,000 burn-in iterations and 10,000 Markov Chain Monte Carlo (MCMC).

To increase the number of subpopulations present in the clones and decrease the possibility of erroneous correlations, the K-value was chosen between 1 and 10 (Gupta et al., 2014). The K-value with the highest likelihood served as the benchmark to establish an appropriate population size for the dataset. After imputation, DArTseq markers were coded as binary values, with 1 denoting presence and 0 denoting absence.

In accordance with Lipka et al. (2012), linkage disequilibrium analysis was performed using the GAPIT function of R software (). Of the 68,814 polymorphic markers, 63,362 had sites that could be used to determine linkage disequilibrium. For each pair of loci, the squared allele frequency correlations R^2^ at p-values of 0.001 were used to evaluate significant linkage disequilibrium.

### Association mapping

To identify the marker characteristic relationships in the population under low and optimal nitrogen regimes, 63,362 DArTseq-generated SNP markers were employed. Following a mixed linear model (MLM) approach that considers both kinship and population structure, association mapping was carried out on traits related to yield and nitrogen use efficiency, such as plant vigor, leaf retention, leaf nitrogen and chlorophyll, stay green ability, dry root yield, fresh root yield, harvest index, and dry matter content (Lipka et al., 2012; Gupta et al., 2014). We determined whether DArT markers were significant at a critical value of 1%. This method is more accurate and less likely to produce spurious marker-trait connections (MTAs) (Gupta et al., 2014).

## Results

### Phenotyping variation across genotypes in two nitrogen regimes

**Figure 1:**
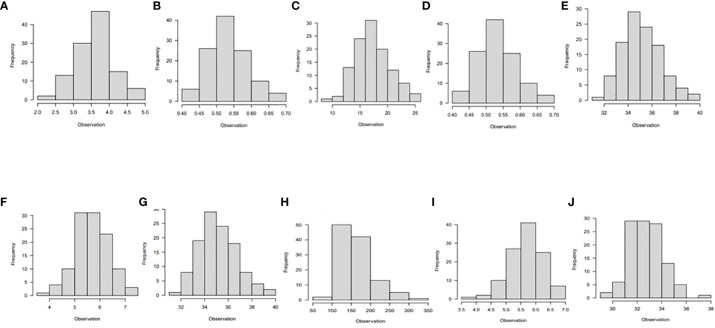
Histogram showing phenotypic distribution of the following traits; **(A)** vigor, **(B)** leaf nitrogen, **(C)** stay-green ability, **(D)** leaf retention, **(E)** chlorophyll, **(F)** fresh root yield, **(G)** dry root yield, **(H)** dry matter content, **(I)** harvest index, and **(J)** nitrogen use efficiency for 265 cassava genotypes evaluated under optimum nitrogen regime in six environments in Nigeria.

**Figure 2:**
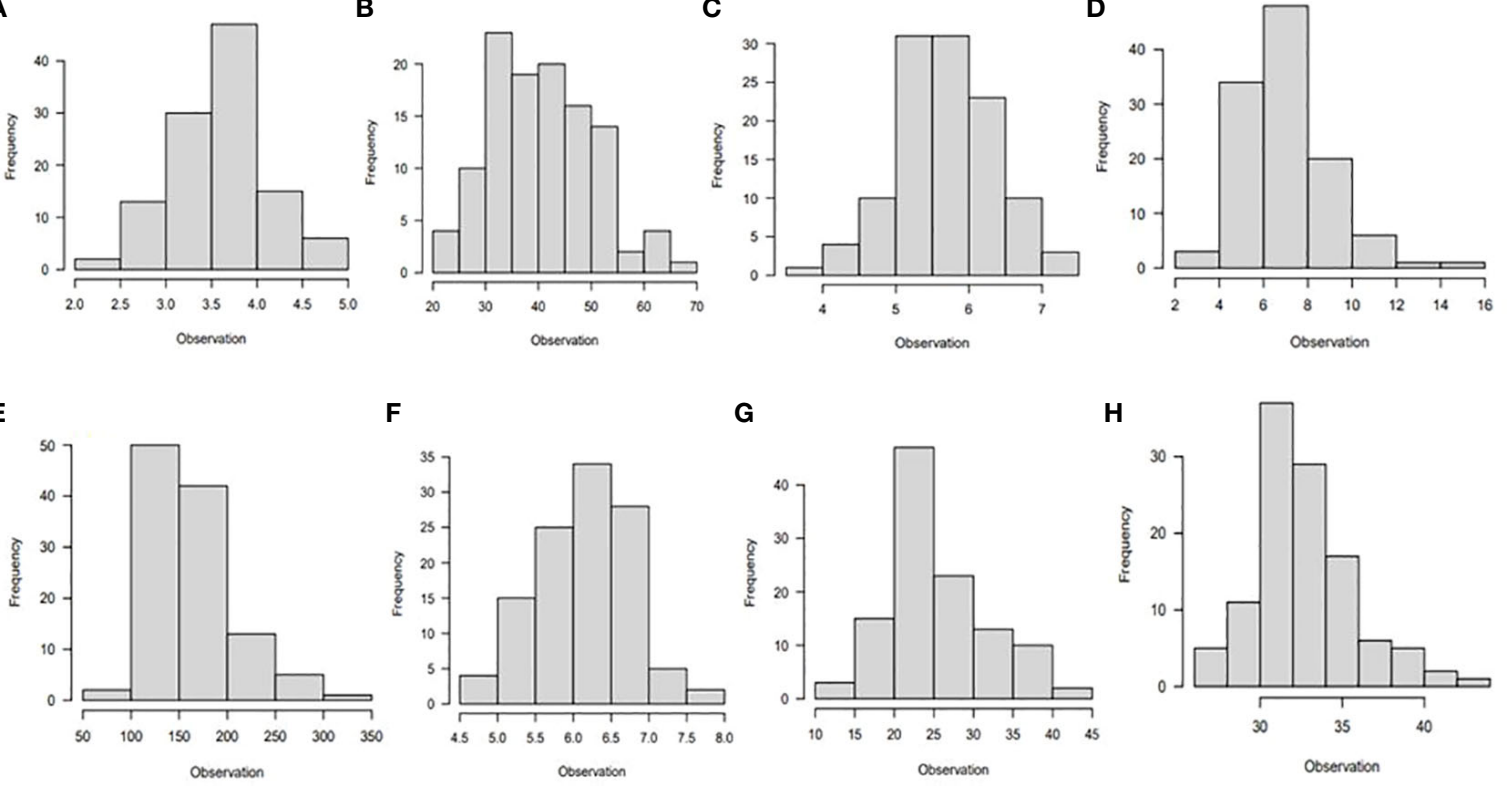
Histogram showing phenotypic distribution of the following traits: **(A)** leaf nitrogen, **(B)** leaf retention, **(C)** chlorophyll, **(D)** fresh root yield, **(E)** dry root yield, **(F)** dry matter content, **(G)** harvest index, and **(H)** nitrogen use efficiency for 265 cassava genotypes evaluated under low nitrogen regime in six environments in Nigeria.

The majority of the traits studied had a normal distribution, as shown by the phenotypic association of the diverse panel averaged across the six different environments (**Figures 1**, **2**). A few traits were slightly skewed towards the tail. A normally distributed random variable has a continuous bell-shaped distribution that is symmetrical about the mean. If a variable with a normal distribution is standardized, the result will be a variable with mean (µ) = 0 and standard deviation (σ) = 1. Typically, a normal distribution is determined by its mean and variance.

### Population structure and kingship matrix

**Figure 3:**
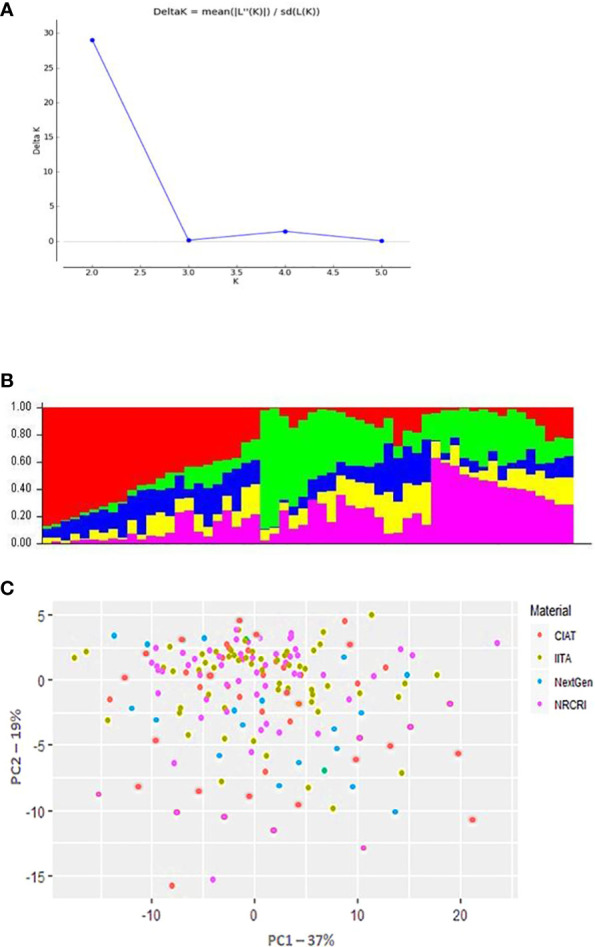
Population structure of 265 cassava genotypes based on 16,362 DArTseq-derived SNP markers. **(A)** Delta K probability values for the five clusters. **(B)** Five clusters, represented by red, green, and yellow. **(C)** Principal Component Analysis (PCA) Plot of PCI against PC2 illustrating population structure in the cassava (*Manihot esculent*) diversity panel genotyped with the SNP markers. The CIAT, IITA, Next-Gen, and NRCRI origins of *M. esculenta* are represented by the red, green, blue, and purple circles, respectively.

The population structure analyses revealed a delta K probability value for five clusters of 265 cassava clones (**Figure 3A**), which suggests that the population could be structured into five clusters, as represented by the red, green, and yellow colors (**Figure 3B**). The 265 diverse cassava genotypes in the population formed a clear population structure and were assembled into two clusters and five subclusters (**Figures 3A, B**). The various colored segments represent the percentage of membership of each genotype in the corresponding clusters. With 27.5% of the population, Cluster 2 had the highest membership, while Cluster 5 had the lowest, at only 12% while the other groups averaged around 15%, Clusters 4 and 5 had the largest level of heterozygosity, resulting in a mean distance within group of 0.61. Principal component analysis (PCA) was used to display information on where the diverse cassava accessions used in this study are from geographically as well as how they are clustered (**Figure 3C**).

**Figure 4:**
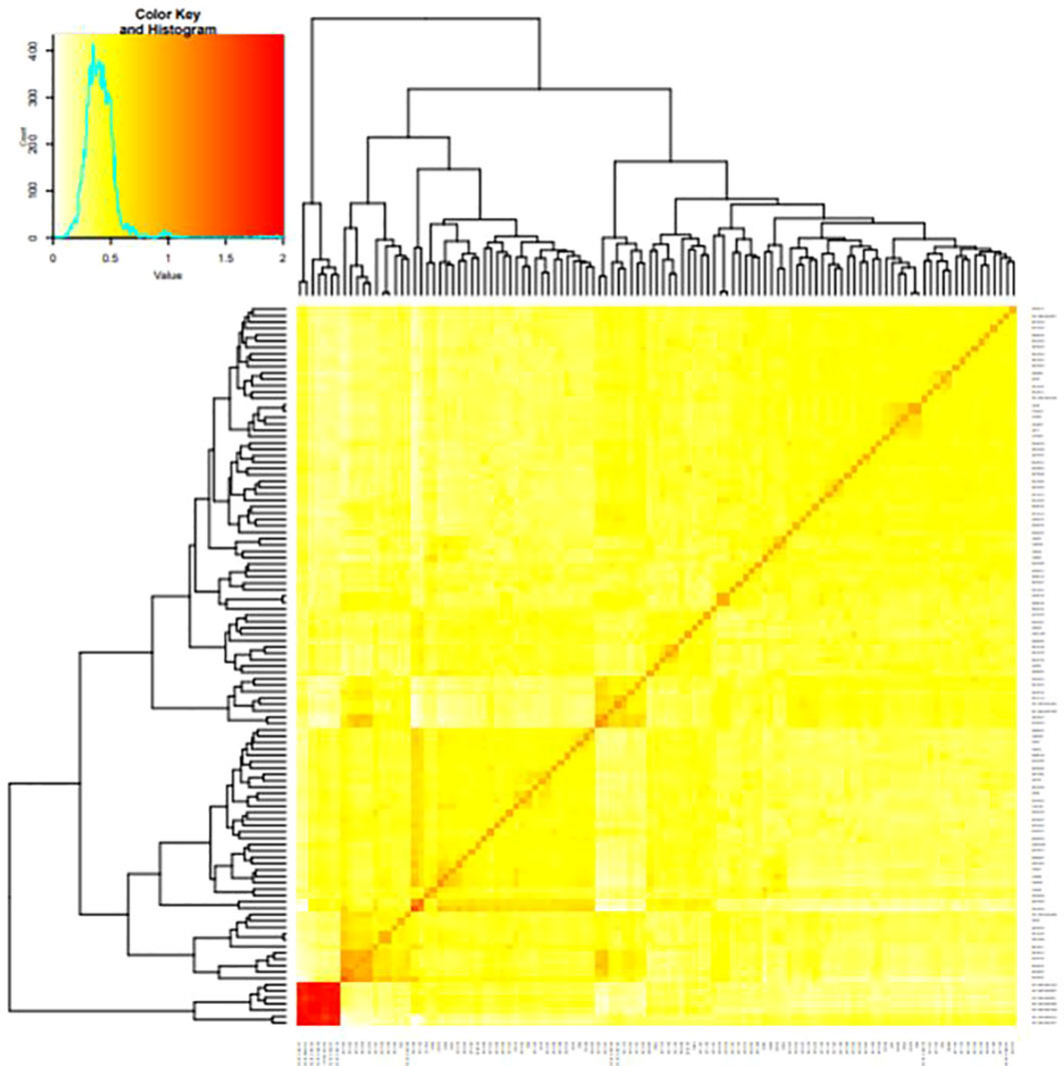
A heat map of the kinship matrix was created to indicate the relationship between the 68,814 SNP markers used in the current study.

The clusters’ average fixation index (Fst) ranged from 0.01 to 0.88. The degree of kinship between the tested clones was a significant factor influencing the GWAS. A kinship value of 0.5 was present in approximately 99% of the pairwise kinship comparisons among the 265 genotypes, demonstrating the unrelatedness of the clones in the population utilized for GWAS. Low levels of relatedness were also seen in the kinship heatmap (**Figure 4**) produced by the vanRanden algorithm in the association panel of the “GAPIT” basic scenario. Low levels of relatedness among the examined clones were further confirmed using a heatmap, where the count of kinship values peaked at zero. In addition, the average genetic distance among the 84,255 pairwise comparisons was 0.3115, with genetic distances ranging from 0.004 to 0.3390. The degree of genetic variation in this study association panel may be shown by the fraction of pairwise comparisons with values higher than 0.3 (14.95%) and with values higher than 0.2 (more than 99%).

### Linkage disequilibrium and genetic distance among genotypes

**Figure 5:**
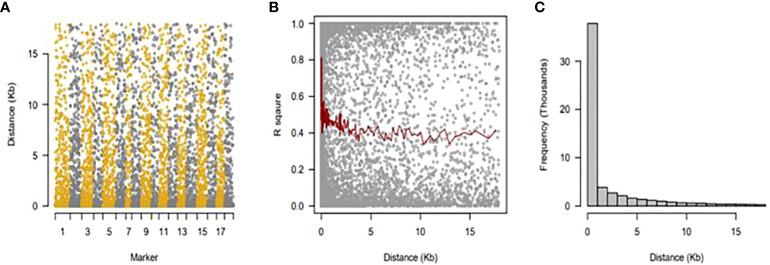
Linkage disequilibrium (LD), decay over distance. **(A)** Sliding windows with 100 nearby genetic markers. **(B)** The average of 10 nearby markers is represented by a red line. **(C)** There was a negative correlation between LD and physical distance (r = −0.14).

LDs were determined on sliding windows with 100 nearby genetic markers (**Figure 5A**). Each dot indicates a pair of distances between two markers on the window and the squared correlation coefficient. The average of the ten nearby markers is represented by the red line (**Figure 5B**). Using linkage disequilibrium analysis, 10,164 loci pairings within a physical range of up to 162,831 bp were found. Only 9.02% of the locus pairs had R^2^ values between 0 and 1, and only 0.43% were completely out of equilibrium. Linkage disequilibrium decreased between distant pairs of markers, and there was a negative correlation between LD and physical distance (r = −0.14). (**Figure 5C**).

### Estimation of nitrogen use efficiency in cassava using BLUP selection index under contrasting N regime

**Figure 6:**
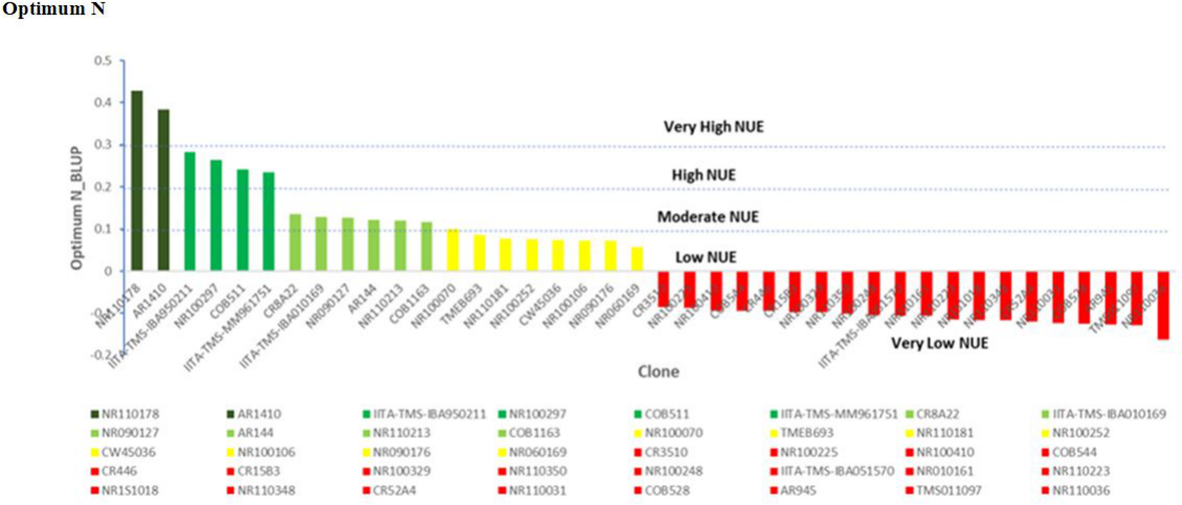
Nitrogen use efficiency (NUE), by genotype and optimum N treatment. Efficiency values are inherently unitless but are based on mass. NUE, units of tuber dry matter produced per unit of nitrogen applied to the field.

Under optimum N treatment, the BLUP selection index identified two genotypes, NR110178 and AR1410, which were very high in nitrogen utilization and most efficient, while four genotypes, IITA-TMS-IBA950211, NR100297, COB511, and IITA-IBA-MM961751, were observed to have high nitrogen use efficient test clones, and six genotypes, CR8A22, IITA-IBA010169, NR090127, AR144, NR110213, and COB1163, were identified as moderately in utilizing N efficiently. It is important to note that the clones identified as very high, high, and moderate outperformed all the checks used in this study. Low NUE test clones were equally identified, including NR100070, TMEB693, NR110181, NR100252, CW45036, NR100106, NR090176, and NR060169 (**Figure 6**).

**Figure 7:**
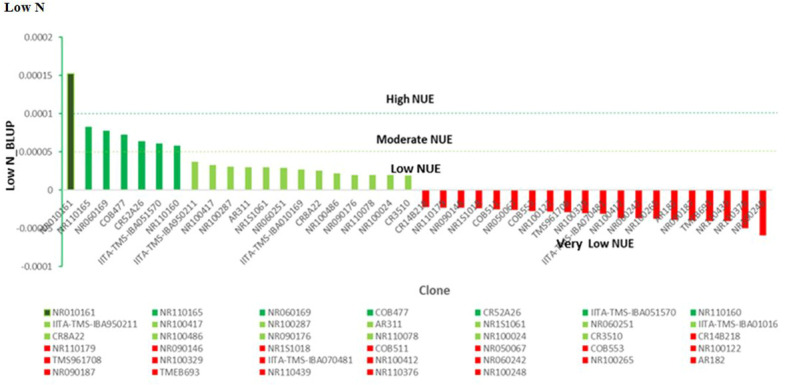
Nitrogen use efficiency (NUE), according to genotype and low-N treatment. Efficiency values are inherently unitless but based on mass. NUE, units of tuber dry matter produced per unit of nitrogen applied to the field.

Under high-N conditions, nitrogen stress was not a factor, since the optimum nitrogen regime made available an exogenous excess of nitrogen; therefore, the genotypes that captured the most nitrogen from their surrounding soil environment were the most N-efficient. Similarly, under the low nitrogen treatment, the test clones were classified into four groups: high, moderate, low, and very low NUE. One genotype (NR010161) showed a high response (substantial yield under low nitrogen conditions) and could be described as having high nitrogen use efficiency. Six genotypes, NR110165, NR060169, COB477, CR542A26, IITA-TMS-IBA051570, and NR110160, were classified as having moderate NUE, whereas IITA-TMS-IBA950211, NR100417, NR100287, AR311, NR1S1061, NR060251, IITA-TMS-IBA010169, CR8A22, NR100486, NR090176, NR110078, NR100024, and CR3510 were classified as having low nitrogen use efficiency (**Figure 7**). The identified genotypes with high and moderate nitrogen use efficiency (NUE) also had high dry matter content, which is an important quality attribute required by consumers, processors, and industries (**Table 4**).

**Table 4:** Yield and dry matter content of the identified high NUE clones across N regimes.

Clones	N regime	Fresh root yield (t/ha)	Dry matter content (%)
NR110178	Optimum N	64.67	37.02
AR1410	Optimum N	60	36.99
IITA-TMS-IBA950211	Optimum N	59.33	36.34
NR100297	Optimum N	58.67	35.02
COB511	Optimum N	56.33	34.98
IITA-IBA-MM961751	Optimum N	54	33.03
CR8A22	Optimum N	51	33.01
IITA-IBA010169	Optimum N	50.67	30.37
NR090127	Optimum N	56.33	30.16
AR144	Optimum N	51	29.77
NR110213	Optimum N	42	26.85
COB1163	Optimum N	41.67	22.39
NR100070	Optimum N	40	22.15
TMEB693	Optimum N	39.33	21.9
NR110181	Optimum N	37.33	21.89
NR100252	Optimum N	36.67	21.76
CW45036	Optimum N	34	21.22
NR100106	Optimum N	33.75	21
NR090176	Optimum N	27.5	20.96
NR060169	Optimum N	24.67	19.94
NR010161	Low N	42.33	36.37
NR110165	Low N	37.33	31.11
NR060169	Low N	35.33	30.85
COB477	Low N	33.33	29.03
CR542A26	Low N	31	28.99
IITA-TMS-IBA051570	Low N	30.67	28.03
NR110160	Low N	28.33	27.98
IITA-TMS-IBA950211	Low N	28	27.61
NR100417	Low N	27	27.55
NR100287	Low N	26.67	25.13
AR311	Low N	26	24.96
NR1S1061	Low N	25	24
NR060251	Low N	23.67	23.81
IITA-TMS-IBA010169	Low N	21	22.71
CR8A22	Low N	20.67	22.61
NR100486	Low N	19	22.39
NR090176	Low N	18.67	22.15
NR110078	Low N	17	21.9
NR100024	Low N	16.7	21.89
NR100024	Low N	14	21.76
CR3510	Low N	13.67	21.22

### SNP marker-trait associations under optimum and low nitrogen regimes

**Figure 8:**
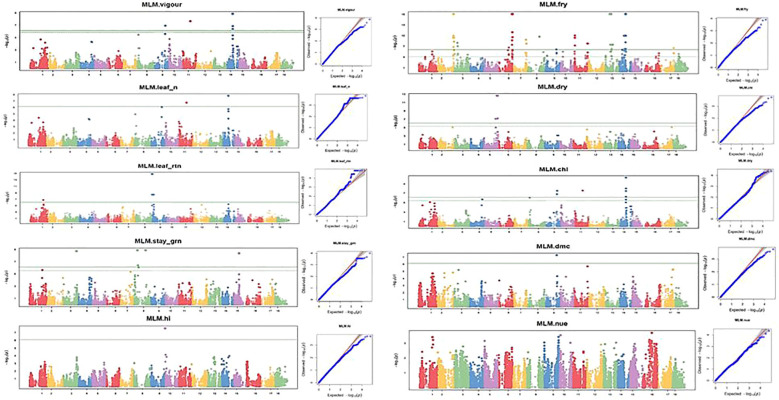
Manhattan and QQ plots for nitrogen use efficiency, fresh root yield, and other related traits evaluated under the optimal nitrogen regime. The horizontal lines in the Manhattan plots show the threshold p-value at the Bonferroni cutoff point of 0.05.

**Figure 9:**
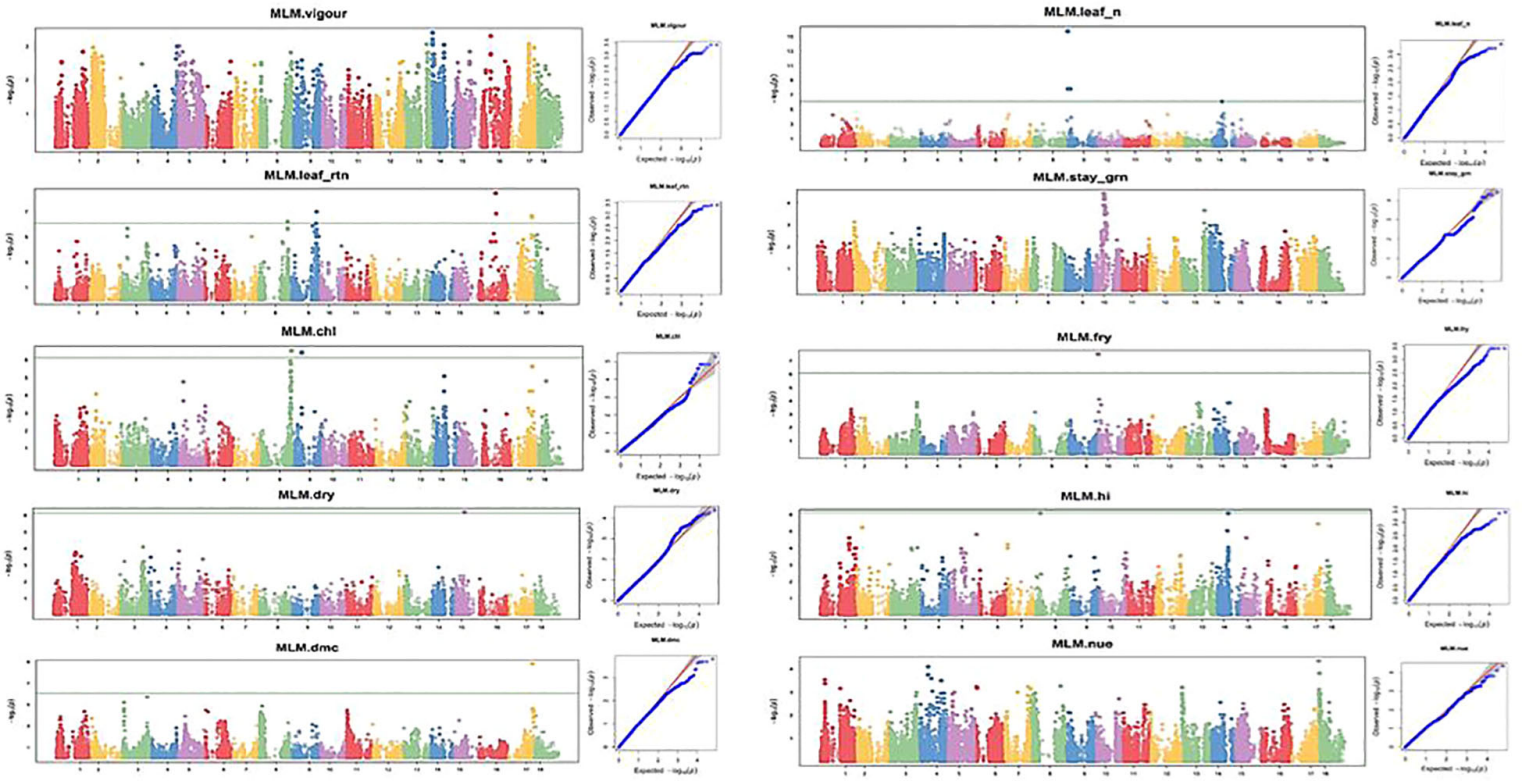
Manhattan and QQ plots for nitrogen use efficiency, fresh root yield, and other related traits evaluated under a low-nitrogen regime. The horizontal lines at Manhattan plots show the threshold p-value at Bonferroni cutoff point of 0.05.

Ten nitrogen use efficiency and yield-related traits (plant vigor, leaf nitrogen content, stay-green ability, leaf retention, chlorophyll, fresh root yield, dry matter content, harvest index, dry root yield, and nitrogen use efficiency) were subjected to GWAS using 68,814 DArTseq-derived SNP markers. Of 68,814 SNP markers, 17 and 35 SNPs with positive associations were detected under low and optimum N regimes, respectively, at a Bonferroni significance level of 5% (**Tables 5**, **6**; **Figures 8**, **9**).

**Table 5:** Summary of SNPs with genome-wide association significance for nitrogen use efficiency and other yield related traits in cassava evaluated under optimum nitrogen regime.

Traits	SNP	Chr	Position	P-value	MAF	Effect
Dry matter content	S9_22496452	9	22496452	4.88E−08	0.027027	−47.9642
Stay-green ability	S8_7913539	8	7913539	1.34E−09	0.00885	12.56518
Stay-green ability	S8_24329904	8	24329904	1.34E−09	0.00885	12.56518
Stay-green ability	S3_26841552	3	26841552	1.96E−09	0.039823	−5.91146
Stay-green ability	S15_10575969	15	10575969	4.35E−09	0.00885	12.29413
Stay-green ability	S8_8865498	8	8865498	3.98E−07	0.017699	7.342439
Stay-green ability	S8_10210834	8	10210834	8.75E−07	0.084071	−6.2704
Stay-green ability	S1_25918531	1	25918531	2.38E−06	0.199115	−4.57286
Leaf retention	S9_937988	9	937988	1.55E−15	0.066372	31.9485
Leaf retention	S9_1036785	9	1036785	3.84E−09	0.070796	16.9836
Leaf retention	S9_1041234	9	1041234	3.84E−09	0.070796	16.9836
Leaf retention	S1_25742358	1	25742358	1.75E−07	0.199115	9.911374
Harvest index	S10_839016	10	839016	1.17E−07	0.080716	−206.211
Dry root yield	S9_937988	9	937988	3.5E−15	0.066372	61.15805
Dry root yield	S9_901738	9	901738	3.08E−08	0.070796	−30.6138
Dry root yield	S9_1041231	9	1041231	3.08E−08	0.070796	−30.6138
Chlorophyll	S14_11921988	14	11921988	4.76E−11	0.00885	197.8183
Chlorophyll	S14_12098734	14	12098734	9.77E−09	0.013274	140.6425
Chlorophyll	S11_15798343	11	15798343	2.67E−08	0.013274	133.1361
Chlorophyll	S9_22529787	9	22529787	2.89E−08	0.013274	133.1071
Chlorophyll	S14_12020725	14	12020725	3.17E−08	0.013274	132.1522
Chlorophyll	S9_22137757	9	22137757	1.53E−07	0.044248	59.97344
Chlorophyll	S14_12020017	14	12020017	5.37E−07	0.017699	104.009
Chlorophyll	S8_3632389	8	3632389	8.99E−07	0.017699	102.6703
Chlorophyll	S4_24203751	4	24203751	1.92E−06	0.017699	98.53843
Vigor	S14_11921988	14	11921988	1.39E−09	0.00885	15.81581
Vigor	S11_15798343	11	15798343	2.18E−08	0.013274	11.83636
Vigor	S9_22529787	9	22529787	1.18E−07	0.013274	11.14026
Vigor	S14_12098734	14	12098734	1.41E−07	0.013274	11.25032
Vigor	S14_12020725	14	12020725	3.94E−07	0.013274	10.56758
Fresh root yield	S10_839016	10	839016	3.08E−08	0.013393	−74.9993
Leaf nitrogen	S14_11921988	14	11921988	1.52E−08	0.00885	8.815767
Leaf nitrogen	S11_15798343	11	15798343	1.79E−07	0.013274	6.595141
Leaf nitrogen	S9_22529787	9	22529787	8.84E−07	0.013274	6.180676
Nitrogen use efficiency	S16_17634768	16	17634768	0.125	0.999933	0.335264

SNP, single nucleotide polymorphism; Chr, chromosome; MAF, minor allele frequency.

**Table 6:** Summary of SNPs with genome-wide association significance for nitrogen use efficiency and other yield related traits in cassava evaluated under low nitrogen regime.

Traits	SNP	Chr	Position	P-value	MAF	Effect
Chlorophyll	S8_32104664	8	32104664	2.9001E−07	0.026549	239.9744
Chlorophyll	S9_8808026	9	8808026	3.7193E−07	0.048673	189.1112
Dry root yield	S15_10079968	15	10079968	6.3205E−07	0.004425	1.758897
Dry matter content	S17_25823721	17	25823721	1.4986E−09	0.026786	−15.8115
Harvest index	S8_5637808	8	5637808	8.307E−07	0.008929	32.73656
Harvest index	S14_12084316	14	12084316	8.307E−07	0.008929	32.73656
Leaf nitrogen	S9_937988	9	937988	1.9547E−16	0.066372	536.1063
Leaf nitrogen	S9_1036785	9	1036785	1.5067E−08	0.070796	253.515
Leaf nitrogen	S9_1041234	9	1041234	1.5067E−08	0.070796	253.515
Leaf nitrogen	S14_10436457	14	10436457	7.8427E−07	0.017699	150.7853
Fresh root yield	S10_28851	10	28851	3.41E−09	0.033453	5.386841
leaf retention	S16_20566079	16	20566079	2.71E−06	0.172566	0.081463
leaf retention	S9_26696959	9	26696959	6.96E−08	0.234513	0.150935
leaf retention	S16_52130	16	52130	2.39E−07	0.084071	0.07379
leaf retention	S18_24315867	18	24315867	2.98E−08	0.482301	−0.04653
leaf retention	S8_29315873	8	29315873	1.55E−08	0.057522	0.174654
Nitrogen use efficiency	S18_114838	18	114838	4.47E−05	0.09375	−0.63048

SNP, single nucleotide polymorphism; Chr, chromosome; MAF, minor allele frequency.

GWAS analysis for NUE at both optimum and low nitrogen regimes revealed non-marker trait associations (MTAs) for the total number of SNPs deployed in this study, but revealed significant SNPs at GWAS thresholds of −log(P) = 3 and 4 for optimum and low nitrogen regimes, respectively. However, the association results for the other nine traits under the optimum nitrogen regime were found at a P-value<0.001, showing putative gene loci for each trait. Manhattan plots for vigor, leaf nitrogen content, leaf retention, dry matter content, chlorophyll, dry root yield, stay-green ability, fresh root yield, and harvest index were obtained from GWAS to explain the positive associations (−log_10_ (p-value) >6) (**Figures 8**, **9**). Association results for traits under the low nitrogen regime revealed marker trait associations (MTAs) for all the traits under study, except for plant vigor and stay-green ability, although significant SNPs were detected at a GWAS threshold of −log(P) = 3 and 4 for vigor and stay green ability, respectively.

GWAS analysis for the optimum nitrogen regime using multiple loci mixed linear model as described by Segura et al. (2012) identified 5, 3, 7, 4, 9, 1, 3, 1, 1, and 1 QTL that were significantly associated with variation in plant vigor, leaf nitrogen, stay-green ability, leaf retention, chlorophyll, dry matter content, fresh root yield, dry root yield, harvest index, and nitrogen use efficiency, respectively. In the low nitrogen regime, GWAS analysis using a multi-linear mixed model identified four, five, two, one, one, one, two, and one significant SNPs found to be linked with variation in leaf nitrogen, leaf retention, chlorophyll, fresh root yield, dry root yield, dry matter content, harvest index, and nitrogen use efficiency, respectively (**Figure 9**).

A quantile–quantile (Q–Q) plot was constructed for each trait under the high- and low-nitrogen regimes to validate their normal distribution (**Figures 8**, **9**). For the optimum nitrogen regime, variation in plant vigor was found to be associated with three loci, one on Chromosome 9, one on chromosome 11, and three on chromosome 14, at a 5% Bonferroni correction. The most significant locus was observed on chromosome 14 and was tagged with marker S14_11921988 (*P* = 1.39E−09) (**Table 5**). Significant SNPs distributions for leaf nitrogen across chromosomes were identified in two loci on Chromosomes 11 and 14 under both low and optimum nitrogen regimes, highlighting the fact that these two markers contributed to the proportion of phenotypic variance.

Variation in stay-green ability in the optimum nitrogen regime was found to be associated with seven loci: one on Chromosome 1, one on Chromosome 3, four on Chromosome 8, and one on Chromosome 15. No significant SNP was above the threshold level in the low nitrogen regime, probably due to nitrogen deficiency in this environment. GWA analysis for leaf retention was significant for both optimum and low nitrogen regimes on Chromosome 9, which accounted for the majority of the variation observed in the traits, S9_937988 (*P* = 1.55E−1) and S9_26696959 (*P* = 6.96E−08), respectively. Similarly, Chromosome 8 had an SNP detected under low and optimum nitrogen regimes, such as S8_3632389 (P = 8.99E−07) for optimum N and S8_32104664 (P = 2.9001E−07) for the low N regime. In the current GWA analysis, a high number of significant SNPs were identified for fresh root yield under the optimum nitrogen regime.

Thirty-one loci of which seven were on Chromosome 2, six on Chromosome 6, two on Chromosome 7, four on Chromosome 11, five on Chromosome 13, and one each on Chromosomes 8 and 9. However, a single marker on chromosome 10 was found to be significantly associated with fresh root yield under low nitrogen regime. GWA analysis of dry root yield identified loci on Chromosomes 4 and 5 under the optimum nitrogen regime, but one locus on Chromosome 15.

Two SNP markers, one on Chromosome 9 and the other on Chromosome 17, for optimum and low nitrogen regimes (**Figures 8**, **9**), were found to be linked to dry matter content. These significant loci were tagged by *SNP S9_22496452* (*P* = 4.88E−08) and *S17_25823721* (P = 1.4986E−09), respectively. Surprisingly, no significant SNP was identified for both optimum and low N regimes for nitrogen use efficiency (NUE) as a trait but revealed top peaks close to the threshold level, signifying loci harboring candidate genes coding for the variability observed for nitrogen use efficiency among SNPs. Marker availability for different traits is fundamental to simultaneously improving two or more traits. In this study, SNPs common to the majority of traits were identified under both low and optimum nitrogen conditions.

### Putative candidate genes

The position of the highly significant SNP markers was explored by subjecting them to fine mapping and a BLAST search on the National Center for Biotechnology Information (NCBI) Genome Viewer v6.0, to annotate genomic regions and detect nearby putative candidate genes associated with nitrogen use efficiency. Putative genes within the significant SNP region were searched for due regard to the significant SNPs position flanking the right and left. Using the databases of the European Molecular Biology Laboratory-European Bioinformatics Institute (EMBL-EBI) and Universal Protein Resource (UniProt), the functions of genes linked to the identified SNPs were discovered. Genomic regions harboring most of the SNPs found to be significant with high peaks were further probed to isolate genes with putative functions using the National Center for Biotechnology Information (NCBI genome information for reference and representative genome assembly *M. esculenta*_v8). In total, 52 putative coding genes were identified that were associated with significant SNPs for both optimum and low nitrogen regimes (**Tables 5**, **6**). These candidate genes have been found to be involved in abiotic stress tolerance in plants, including nutrient deprivation.

The candidate gene *LOC110620977 MANES_08G064200v8* coding for stay-green ability under optimum N and annotated as V-type proton ATPase subunit H was found within SNP S8_8865498 on Chromosome 8, which is associated with an ATP-driven enzyme that uses the major active transport of H+ to convert ATP hydrolysis energy into electrochemical potential differences of protons across various biological membranes.

A putative gene (*LOC110600224 MANES_14G137400*) encoding the ubiquitin-like domain-containing protein CIP73 was found to be very close to the significant SNPs identified for chlorophyll under the low-N regime on Chromosome 14. A potential candidate gene (*LOC110600354 MANES_14G130700*) encoding for NADH-cytochrome b5 (CB5s) reductase-like protein in leaf nitrogen revealed that hormone signaling regulators or plant stress response, catalytic enzymes such as hydroxylases, transporters, P450 monooxygenases, desaturases, reductases, and others all engage strongly with CB5s. Such diverse interactions suggest that plant CB5s may perform roles other than those of simple electron carriers in regulating enzymatic reactions and metabolic processes.

### Multiple sequence alignment

**Figure 10:**
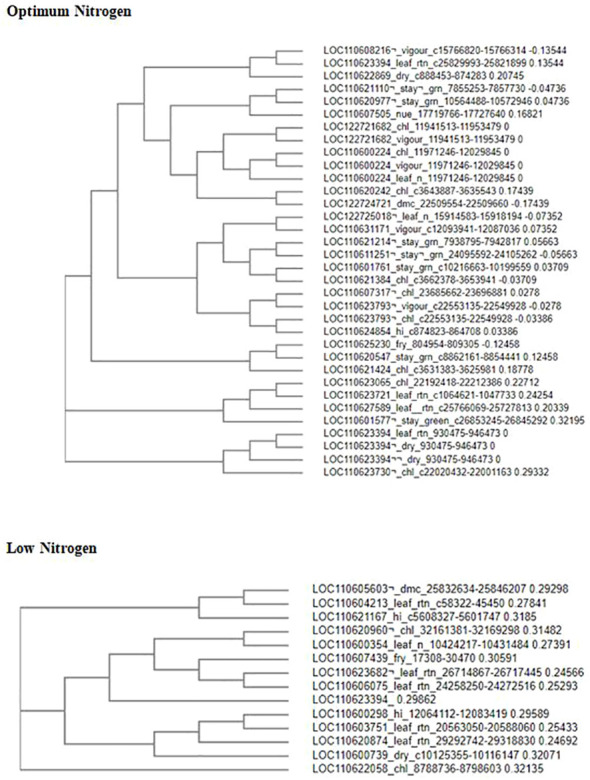
Phylogenetic tree showing the distribution and relatedness of protein coding genes for nitrogen use efficiency and other related traits in cassava: vigor, leaf retention, stay green, leaf nitrogen, chlorophyll, fresh root yield, dry matter content, dry, and harvest index, Sequences were aligned with cluster omega in multiple sequence alignment.

The Clustal Omega website of the European Molecular Biology Laboratory for European Bioinformatics Institute (EMBL-EBI), a genomic website, was used for multiple alignments of putative candidate genes to plot the phylogenetic tree analysis. This tool was used to identify inherent relationships and common patterns between genes. It shows the sequence alignment of three or more biological sequences, usually DNA, RNA, or proteins. The alignments were generated and analyzed using computational algorithms. It uses seeded guide tree techniques to generate alignments between three or more sequences. In the present study, the alignment of divergent protein sequences was computed by assigning individual weights to each sequence in a partial alignment in order to downweight near-duplicate sequences and up-weight the most divergent sequences (**Figure 10**). Similarly, amino acid substitution matrices vary at different alignment stages, according to the divergence of the sequences to be aligned. Neighbor-joining tree analysis explained how the associated candidate genes were biologically linked. The results showed six and four clusters of gene association for the optimum and low-nitrogen regimes, respectively. Most of the genes shared common associations and linkages.

## Discussion

This study showed that all types of efficiency were higher under the low-N regime, as utilization was the major driver of yield in a low-N environment. This concurs with earlier studies on potatoes and other crops (Hewitt, 1963; Novoa and Loomis, 1981). The N level determines the relative importance of two NUE components. However, NUE in maize seems to be relatively constant across N levels (Gallais and Coque, 2005). However, discrepancies in NUE under low-N and high-N regimes have been observed in other crops, including winter wheat (*Triticum aestivum* L.) and spinach (Biemond, 1995; Gaju et al., 2011).

The names “high-NUE genotypes” and “low-NUE genotypes,” according to a number of studies, refer to genotypes that produce high yields at levels of high and low N, respectively (Ladha et al., 2005). Some experts define high-NUE genotypes as those with the ability to produce significant yields while surviving in low-N environments (He et al., 2014; Quan et al., 2017). The two benefits of screening for high-NUE genotypes at high N levels are their high fertilizer tolerance and facilitation of high yields under high N conditions. Not all genotypes with high NUEs were found as a result of selection in high-N environments. Therefore, low-nitrogen conditions should be used for selection in breeding operations.

A total of 68,814 SNPs at various densities were distributed along the 18 chromosomes, of which 52 SNPs were positively associated with nitrogen use efficiency in cassava and other yield-related traits. The average polymorphic information content (PIC) value (0.60) obtained in the present study, is much higher than that reported by Udoh et al. (2017), as well as that reported in several other studies (Simko et al., 2012; Zhang et al., 2016; Adewale et al., 2020). This revealed the informativeness and high polymorphism of the SNP markers used in this study.

The population structure was estimated to elucidate individual relatedness in association studies, which is a prerequisite for eliminating or reducing false associations (Yu and Buckler, 2006). The analysis revealed the delta K probability value of the five clusters, which is indicative of the diversity of the cassava clones used in the study. The genotypes formed a clear population structure and were grouped into two clusters and five subclusters (**Figures 3A, B**), and the various colored segments estimated the proportion of each genotype’s membership in the respective clusters.

Quantile–quantile (Q–Q) plot was used to determine model fitness for the association analysis, plotting the observed versus expected P-values under the null hypothesis that there is association existing between an SNP and the phenotype. This has become imperative because GWAS has been widely deployed to find genetic variations that are statistically related to a particular attribute by examining hundreds of thousands of them across numerous genomes. However, complex genetic architecture and polygenic traits, on the other hand, can produce false positive and indirect associations in GWAS. The results revealed that for all traits studied, the majority of the Q–Q plot points were aligned on the diagonal line, suggesting that population structure and relative kinship-based spurious allelic correlations were significantly decreased. This finding is consistent with that of a previous study by Adewale et al. (2020).

The identification of major alleles and protein-coding genes directly associated with nitrogen use efficiency and other yield-related traits in cassava is imperative for the development of cassava genotypes that would respond to low-nitrogen soils through marker-assisted breeding. These GWA results provide insights into the genetic basis of NUE and other yield-related traits in cassava. A total of 52 significant SNP markers were associated with vigor, leaf nitrogen, stay green ability, leaf retention, dry matter content chlorophyll, fresh root yield, dry root yield, and harvest index at the threshold of –log(_P_) = 6.

Publicly available Genome Viewer v6.0 (NCBI) was used for annotation of candidate genes surrounding or adjacent to the significant SNP markers. Some candidate genes play an important role in plant growth, hormonal signalling, and abiotic stress tolerance, including drought, salinity, and nutrient deficiency. A putative gene (LOC110600224 MANES_14G137400) encoding the ubiquitin-like domain-containing protein CIP73 (**Table 7**), was found to be very close to the significant SNPs identified for chlorophyll under the low nitrogen regime on chromosome 14. This candidate gene plays an important role in growth, hormonal signalling, and abiotic stress tolerance in plants, such as salinity, heat stress, drought, and nutrient deprivation (Sharma et al., 2016). Similarly, under optimum nitrogen regime, a candidate gene (LOC110607439 MANES_10G007200) with the gene function protein reticulate-related 6, chloroplast (**Table 8**), associated with proteins of the reticulate family functionally link photoperiodic growth, amino acid homeostasis, and reactive oxygen species metabolism during Arabidopsis leaf growth (Perez-Perez, 2013). According to Xiao-Hong et al. (2014), to maintain an electrochemical H+-gradient across the tonoplast, stimulating Na+ sequestration into the central vacuole, and improving salt stress tolerance in plants, V-type proton ATPase subunit H is essential. To further understand the potential roles of these putative genes in cassava, more research is required, which has not been thoroughly researched in cassava. The putative genes identified through GWAS in this study, particularly those with significant associated markers for the traits under consideration, can serve as crucial guides for the creation of functional markers. By utilizing marker-assisted selection, these gene-based functional markers may enhance the nitrogen utilization efficiency and other yield-related parameters in cassava.

**Table 7:** Candidate genes co-located with significant SNPs associated with nitrogen use efficiency and other related traits in cassava evaluated under optimum nitrogen regime.

Traits	Chr	Position	Candidate genes	Gene description
Dry matter content	9	22496452	LOC122724721	small nucleolar RNA R71
Stay-green ability	8	7913539	LOC110621214 MANES_08G061400v8 LOC110621435 MANES_08G061350v8	Probable mediator of RNA polymerase II transcription subunit 26b zinc finger MYM-type protein 1-like
Stay-green ability	8	24329904	LOC110621110 MANES_08G088800v8	UPF0161 protein At3g09310
Stay-green ability	3	26841552	LOC110611251 MANES_03G140200v8	mitochondrial import inner membrane translocase subunit TIM50
Stay-green ability	15	10575969	LOC110601577 MANES_15G131800v8	phosphoglucomutase, cytoplasmic
Stay-green ability	8	8865498	LOC110620977 MANES_08G064200v8	V-type proton ATPase subunit H
Stay-green ability	8	10210834	LOC110620547 MANES_08G067100v8	pyrophosphate–fructose 6-phosphate 1-phosphotransferase subunit alpha
Stay-green ability	1	25918531	LOC110601761	glutamate–cysteine ligase, chloroplastic-like
Leaf retention	9	937988	LOC110623394 MANES_09G003100v8	hydroxymethylglutaryl-CoA lyase, mitochondrial
Leaf retention	9	1036785	LOC110623394 MANES_09G003100v8	hydroxymethylglutaryl-CoA lyase, mitochondrial
Leaf retention	9	1041234	LOC110623721 MANES_09G003700v8	rhomboid-like protein 20
Leaf retention	1	25742358	LOC110627589 MANES_01G061050v8	lysine-specific demethylase 5C
Harvest index	10	839016	LOC110624854 MANES_10G007200v8	protein RETICULATA-RELATED 6, chloroplastic
Dry root yield	9	937988	LOC110623394 MANES_09G003100v8 LOC110622869 MANES_09G002900v8	hydroxymethylglutaryl-CoA lyase, mitochondrial putative SWI/SNF-related matrix-associated actin-dependent regulator of chromatin subfamily A member 3-like 1
Dry root yield	9	901738	LOC110622869 MANES_09G002900v8	putative SWI/SNF-related matrix-associated actin-dependent regulator of chromatin subfamily A member 3-like 1
Dry root yield	9	1041231	LOC110623394 MANES_09G003100v8	hydroxymethylglutaryl-CoA lyase, mitochondrial
Chlorophyll	14	11921988	LOC122721682 MANES_14G136856v8	receptor-like protein 13
Chlorophyll	14	12098734	LOC122721682 MANES_14G136856v8	receptor-like protein 13
Chlorophyll	11	15798343	LOC12272501	uncharacterized LOC122725018
Chlorophyll	9	22529787	LOC110623793 MANES_09G085100	glucan endo-1,3-beta-glucosidase 5
Chlorophyll	14	12020725	LOC110600224 MANES_14G137400	ubiquitin-like domain-containing protein CIP73
Chlorophyll	9	22137757	LOC110623065 MANES_09G084600 LOC110623730 MANES_09G083700	SEC14 cytosolic factor sister chromatid cohesion protein PDS5 homolog A
Chlorophyll	14	12020017	LOC110600224 MANES_14G137400	ubiquitin-like domain-containing protein CIP73
Chlorophyll	8	3632389	LOC110620242 MANES_08G037200 LOC110621424 MANES_08G037000 LOC110621384 MANES_08G037300	ABC transporter G family member 22 sucrose-phosphatase 1 calcium-dependent protein kinase 20
Chlorophyll	4	24203751	LOC110607317	uncharacterized LOC110607317
Vigor	14	11921988	LOC122721682 MANES_14G136856v8	receptor-like protein 13
Vigor	11	15798343	LOC110608216	auxin-induced protein 15A-like
Vigor	9	22529787	LOC110623793 MANES_09G085100	glucan endo-1,3-beta-glucosidase 5
Vigor	14	12098734	LOC110631171 MANES_14G137900	110 kDa U5 small nuclear ribonucleoprotein component CLO
Vigor	14	12020725	LOC110600224 MANES_14G137400	ubiquitin-like domain-containing protein CIP73
Fresh root yield	10	839016	LOC110625230 MANES_10G006600	alpha-soluble NSF attachment protein 2
Leaf nitrogen	14	11921988	LOC110600224 MANES_14G137400	ubiquitin-like domain-containing protein CIP73
Leaf nitrogen	11	15798343	LOC122725018	uncharacterized LOC122725018
Leaf nitrogen Nitrogen use efficiency	9 16	22529787 17634768	LOC110623793 MANES_09G085100 LOC110607505	glucan endo-1,3-beta-glucosidase 5 uncharacterized LOC110607505

**Table 8:** Candidate genes co-located with significant SNPs associated with nitrogen use efficiency and other related traits in cassava evaluated under low-nitrogen regime.

Traits	Chr	Position	Candidate genes	Gene annotation
Chlorophyll	8	32104664	LOC110620960 MANES_08G094900	pre-mRNA-splicing factor CWC22 homolog
Chlorophyll	9	8808026	LOC110622058 MANES_09G053000	sorting nexin 1
Dry root yield	15	10079968	LOC110600739 MANES_15G127100	putative 1-phosphatidylinositol-3-phosphate 5-kinase FAB1C
Dry matter content	17	25823721	LOC110605603 MANES_17G059600	ubiquinone biosynthesis O-methyltransferase, mitochondrial
Harvest index	8	5637808	LOC110621167 MANES_08G052600	calcium-transporting ATPase 4, plasma membrane-type
Harvest index	14	12084316	LOC110600298 MANES_14G137700	probable protein phosphatase 2C 27
Leaf nitrogen	9	937988	LOC110623394 MANES_09G003100	hydroxymethylglutaryl-CoA lyase, mitochondrial
Leaf nitrogen	9	1036785	LOC110623394 MANES_09G003100	hydroxymethylglutaryl-CoA lyase, mitochondrial
Leaf nitrogen	9	1041234	LOC110623394 MANES_09G003100	hydroxymethylglutaryl-CoA lyase, mitochondrial
Leaf nitrogen	14	10436457	LOC110600354 MANES_14G130700	NADH-cytochrome b5 reductase-like protein
Fresh root yield	10	28851	LOC110607439 MANES_10G007200	protein RETICULATA-RELATED 6, chloroplastic
leaf retention	16	20566079	LOC110603751 MANES_16G056600	protein NARROW LEAF 1
leaf retention	9	26696959	LOC110623682 MANES_09G089600	zinc transporter 8
leaf retention	16	52130	LOC110604213 MANES_16G000400	jasmonate-induced oxygenase 1
leaf retention	18	24315867	LOC110606075 MANES_18G128000	laccase-3
leaf retention Nitrogen use efficiency	8 18	29315873 114838	LOC110620874 MANES_08G087300 MANES_18G000051v8	mRNA-capping enzyme NADH dehydrogenase subunit 1

## Conclusion

This study is one of the few on nitrogen use efficiency in cassava using a GWAS approach to identify candidate genes associated with NUE and other yield-related traits. A mixed-model derived best linear unbiased prediction selection index was used to estimate nitrogen use efficient genotypes in the optimum and low-nitrogen regimes. Under optimum nitrogen treatment, two genotypes, NR110178 and AR1410, were very high in nitrogen utilization and were the most efficient, while four genotypes, IITA-TMS-IBA950211, NR100297, COB511, and IITA-IBA-MM961751, were observed to have high nitrogen use efficiency. However, under the low nitrogen treatment, one genotype (NR010161) responded very well to the N stress condition and could be described as having high nitrogen use efficiency. Six genotypes were classified as having moderate NUE: NR110165, NR060169, COB477, CR542A26, IITA-TMS-IBA051570, and NR110160. These genotypes identified under low-N conditions are promising genotypes that can be ascribed to being very efficient in N use. A total of 68,814 SNPs were at various densities found scattered along 18 chromosomes, of which 52 were identified as being associated with nitrogen use efficiency in cassava and other yield-related traits. These putative genes identified through GWAS, especially those with significantly associated SNP markers for NUE and related traits, have the potential, if deployed appropriately, to improve nitrogen use efficiency in cassava varieties.

Finally, some of the putative genes *LOC122725018* and *LOC110607317* were discovered on Chromosomes 11and 4. For chlorophyll, *LOC122725018* found in Chromosome 11 for leaf nitrogen, and *LOC110607505* in Chromosome 16 for nitrogen use efficiency under the optimum N regime are uncharacterized genes and have not been reported elsewhere. These are new discoveries; therefore, they significantly contribute to knowledge.

## Data availability statement

The original contributions presented in the study are included in the article/supplementary files, further inquiries can be directed to the corresponding author/s.

## Author contributions

JM: Conceptualization, Data curation, Formal Analysis, Funding acquisition, Investigation, Methodology, Project administration, Resources, Software, Supervision, Validation, Visualization, Writing – original draft, Writing – review & editing. DD: Conceptualization, Methodology, Resources, Supervision, Writing – review & editing. SA: Data curation, Formal Analysis, Investigation, Methodology, Writing – review & editing. DN: Investigation, Methodology, Writing – review & editing. JO: Conceptualization, Funding acquisition, Investigation, Project administration, Resources, Validation, Writing – review & editing. PT: Conceptualization, Investigation, Methodology, Supervision, Writing – review & editing. CE: Conceptualization, Funding acquisition, Investigation, Methodology, Project administration, Resources, Supervision, Writing – review & editing.
